# Implementation of a Low-Carbohydrate Diet Improves the Quality of Life of Cancer Patients – An Online Survey

**DOI:** 10.3389/fnut.2021.661253

**Published:** 2021-08-11

**Authors:** Julia Tulipan, Barbara Kofler

**Affiliations:** Research Program for Receptor Biochemistry and Tumor Metabolism, Department of Pediatrics, University Hospital of the Paracelsus Medical University, Salzburg, Austria

**Keywords:** ketogenic diet, ketosis, online survey, human study, cancer

## Abstract

**Background:** The ketogenic diet (KD), a high-fat low-carbohydrate diet, has gained in popularity in recent years, which is reflected by an increasing number of scientific articles, books, websites, and other publications related to low carbohydrate (LC) diets and KDs. Numerous preclinical studies in different animal models of cancer have examined the effect of KDs on cancer growth, but no large randomized controlled studies or prospective cohort studies are available for human cancer patients. Evidence supporting the use of KDs as an adjunct to traditional cancer therapy has come predominantly from anecdotes and case reports. The first KD clinical trials in patients with glioblastoma revealed good acceptance and a possible anti-tumor effect. Metabolic therapy options such as the KD are not yet part of the standard of care in cancer patients. However, many cancer patients have begun implementing a KD or LC diet on their own. The aim of the present study was to gather information, via an online questionnaire, about how cancer patients go about implementing a KD or LC diet, what resources they rely on, whether they perceive benefits from the diet on quality of life (QoL), and what factors influence feasibility and adherence to the diet.

**Method:** Recruitment of participants was carried out via social media platforms, forums and cooperating physicians (April 2018 through November 2018). To be eligible for the study, participants had to be diagnosed with cancer and on a KD or LC diet at the time of participating in the study or been on a KD or LC diet during cancer treatment. Study participants were asked to fill out an online questionnaire. The questionnaire was divided into four parts and contained a total of 64 questions. The questions were focused on the current health status of the participant, type of cancer, time since diagnosis, and treatment regimen. In addition, questions addressed social support, extent of professional counseling, food preferences and QoL.

**Results:** A total of 96 participants (77 F, 17 M) submitted the questionnaire, of which 94 were included in the final data analysis. Ages ranged between 24 and 79 years (mean 50.1 ± 12.1 years). In 73.4% of the participants, the tumor had not formed metastases at the time of initial diagnosis. Twenty-four (26%) participants had a PET-positive tumor, 8 (9%) a PET-negative tumor, and the remainder (66.0%) did not report a PET scan. Eighty seven percent had undergone surgery in the course of their cancer treatment. The most frequent tumor type was breast cancer, followed by cervical cancer, prostate cancer, colorectal cancer and melanoma. Fifty nine percent of the study participants stated that they followed a KD during cancer therapy, 21% followed a low carbohydrate/high fat (LCHF) diet and 12% followed a low glycemic index (LOGI) diet. Sixty nine percent reported an improvement of QoL because of the diet. Almost half of the study participants sourced their initial information on KDs and LC diets from the internet. We found a significant correlation between weight loss upon implementation of a KD and the extent of overweight (*p* < 0.001). Weight loss in already lean participants was not reported. Overall, 67% of the participants found long-term adherence to the diet to be “easy” and 10.6% described it as being “very easy.” Participants who like fatty foods tended to perceive the diet as being easier to follow (*p* = 0.063).

**Conclusion:** The KD or LC diet improved self-reported QoL in more than two-thirds of study participants. The KD had a normalizing effect on body weight. The majority of the participants rated the diet as easy or very easy to follow long term. There was an obvious gap between patients' desire for professional dietary counseling and what is currently offered by health care providers. In the future, efforts should be made to invest in nutrition experts who are trained in the KD to support cancer patients with implementation of a KD.

## Introduction

Ketogenic diets (KDs) are characterized by high fat intake, moderate-to-low protein consumption, and very low carbohydrate intake (usually <50 g), resulting in an increase of plasma ketone bodies, especially 3-hydroxybutyrate (3HB). Elevated 3HB plasma levels also occur after fasting or physical exercise, leading to mitochondrial respiration rather than glycolysis for energy production. KDs are used as a nutritional therapeutic strategy in certain diseases, including pharmacoresistant epilepsy, GLUT-1 transporter deficiency, and pyruvate-dehydrogenase deficiency (PDHD). Recent research suggests that KDs could be beneficial in the treatment of other diseases, like cancer (1–3).

Most cancer cells display an altered metabolic phenotype characterized by enhanced glycolysis and lower oxidative phosphorylation (4). Thus, a KD which reduces glucose availability to tumor cells while providing ketone bodies as an alternative fuel to normal cells holds promise as an adjunct therapeutic option in cancer patients (5). In principle, a KD should lead to selective starvation of tumor cells, which are unable to adapt to ketone metabolism (6).

To date, there have been numerous preclinical studies of the anti-tumor potential of KDs in different animal models, but no large randomized controlled studies or prospective cohort studies (7, 8). Evidence that KDs could be effective as an adjunct cancer therapy in humans comes largely in the form of anecdotes and case reports. A pilot study published in 2014 showed feasibility and good tolerability of a KD protocol in cancer patients (9). The first clinical trials in patients with glioblastoma showed good acceptance and a possible anti-tumor effect (6, 10–12).

Besides having possible anti-tumor effects, KDs may improve patient quality of life (QoL), by reducing fatigue, reducing chemotherapy side effects, and positively affecting body composition (13–15). In a clinical trial, eleven patients with advanced or metastatic tumors were put on a modified KD (20–40 g of carbohydrates per day). The modified KD was safe and feasible in advanced cancer. QoL was preserved or slightly improved. After 4 weeks, 54.5% of the patients had stable or partially improved disease (16). Klement and Sweeney (17) described six patients who underwent radiotherapy and concurrently consumed a self-administered KD. All patients were followed prospectively with measurements of blood parameters, QoL, body weight and body composition. No adverse diet-related effects were reported. All patients rated the KD as satiating. Weight loss was seen in all patients, but it reached statistical significance only in two. Bioimpedance measurements indicated that weight loss was mainly due to fat loss. Muscle mass was very well preserved. Overall QoL remained stable and all patients reported feeling good on the diet. Five patients with early-stage disease showed tumor regression. One subject with metastatic small cell lung cancer experienced slight progression during three cycles of combined chemotherapy and KD. Tumor progression rapidly accelerated after ending the KD (17).

The KD has gained in popularity, which is reflected by the increasing number of books, websites, scientific articles and other publications dealing with KD in different diseases. Metabolic therapy options such as the KD are not yet part of the standard of care in cancer treatment. Nonetheless, large numbers of cancer patients have been implementing a KD or LC diet on their own. The aim of the present study was to gather information about how patients are implementing a KD or LC diet, what resources they rely on, and what factors influence feasibility and adherence. An additional aim was to gather more information on the subjective effects of the diet on QoL.

## Methods

The study, including the use of the proposed online questionnaire, was approved by the Donauuniversität Krems ethics committee on March 1st, 2018 (EK GZ 26/2015-2018). Inclusion criteria for the participants were that they had been diagnosed with cancer and were on or had been on a KD or LC diet during or after cancer treatment at the date of survey participation. Statistical analysis was carried out in Microsoft Excel and SPSS, version 25.0. For descriptive statistical analysis, we generated contingency tables and determined absolute numbers and relative frequencies.

### Recruitment and Data Security

Recruitment of participants was carried out via the social media platform Facebook. These were mainly closed groups where people exchange their personal experiences regarding the KD and cancer. Additionally, physicians and non-medical practitioners who had indicated that they have patients following a KD or LC diet were contacted and asked to inform their patients about the survey. Participants were asked to fill out an online questionnaire (Supplementary Material); alternatively, a downloadable PDF was available. The online questionnaire was built with Gravity Forms, a WordPress application (http://www.gravityforms.com). All data collected were hosted on a secure server. This guaranteed data safety and anonymized analysis of the data, since every form entry was assigned a unique serial number. Some participants provided their e-mail address if they were interested in participating in a possible follow-up study. In that case, we guaranteed that only staff directly involved in the study would have access to the data. To eliminate duplicates, we checked IP-addresses and excluded forms sent from the same IP-address. For data analysis, submitted questionnaires were excluded if (i) no tumor type was stated, (ii) no dietary changes had been implemented, and/or (iii) cross checking of answers showed major discrepancies and incoherence.

### Study Design and Questionnaire

The questionnaire was divided into four parts and contained a total of 64 questions (Supplementary Material). We asked for information on the disease (*n* = 15), diet and meal frequency (*n* = 18), counseling received (if any) and how information about the diet was obtained (*n* = 8), QoL (*n* = 10), food preferences (*n* = 6), and personal information about socioeconomic status (*n* = 7). Between April 2018 and November 2018, the link to the questionnaire was posted several times to LC diet and KD Facebook groups and sent to physicians and researchers who are working in this area. A total of 96 individuals submitted a questionnaire. Two participants were excluded from the final data analysis. One was excluded because no tumor type was stated, and the other because there were inconsistencies in the responses regarding the date of cancer diagnosis and when the therapy ended.

## Results

### Participant Characteristics

In total, 94 completed questionnaires were included in the final analysis, from 77 women and 17 men. Mean age of the respondents was 50.1 (±12.1) years, median 49.1 years, range 24 to 79 years.

### Tumor Type and Status of the Disease

In 73% of the participants the tumor had not metastasized at the time of initial diagnosis. Twenty-four patients (25.5%) had a PET-positive tumor, 8 patients (8.5%) had a PET-negative tumor, and the remainder (66.0%) did not report a PET scan. Eighty seven percent had surgery during their cancer treatment. The most frequent tumor type was breast cancer (44%), followed by cervical cancer, prostate cancer, colorectal cancer, and melanoma (Table 1).

**Table 1:** Numbers of tumor types among the study cohort.

**Tumor type**	**Total**	**Female**	**Male**
Adenocarcinoma of the endocervical glands	1	1	0
Cervical-CA	7	7	0
Colorectal-CA	5	2	3
Glioma	1	1	0
Testicular cancer	1	0	1
Hodgkin's Lymphoma	3	2	1
Cartilage-CA	1	1	0
Liver-CA	1	1	0
Leukemia	2	1	1
Lung-CA	4	3	1
Breast-CA	43	43	0
Medullary thyroid-CA	2	2	0
Melanoma	5	3	2
Mucinous Adeno-CA	1	1	1
Muscle-CA	1	1	0
Adenocarcinoma	1	1	0
Ovarian-CA	3	3	0
Pancreatic-CA	2	1	1
Plasmacytoma	1	1	0
Prostate-CA	6	0	6
Thyroid-CA	3	3	0
Total	94		

### How the Participants Learned About the KD or LC Diet

We asked the study participants how they first learned about the possibility of a complementary nutritional intervention to the standard cancer therapy. Nearly half (48%) became aware of the KD or LC diet through an article on the internet, 15% obtained the initial information from friends and family, and 12% read about it in a book.

### Characteristics of the Diet Used and Dietary Habits

**Figure 1:**
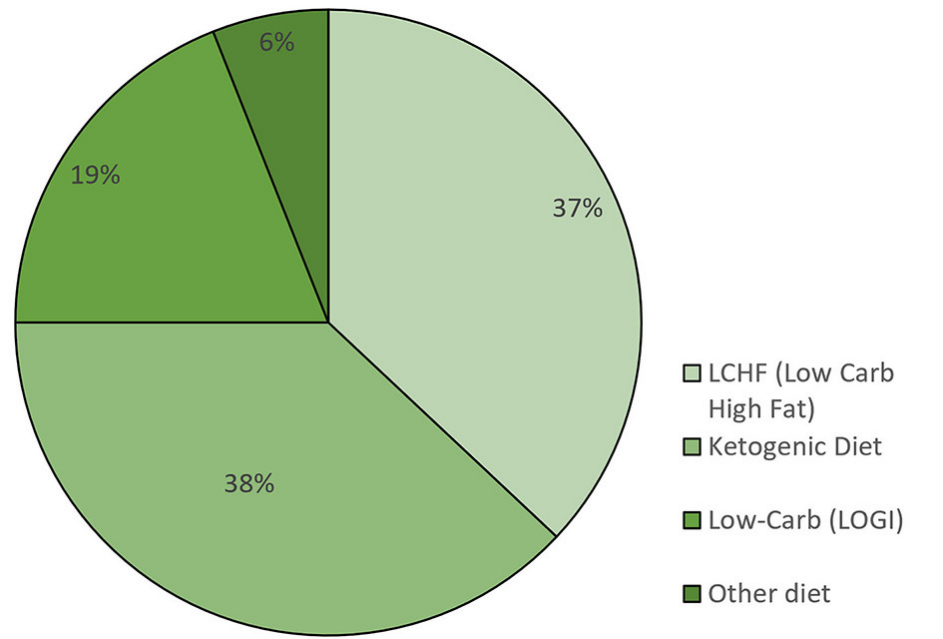
Percentage of participants following a specific diet at the time of filling out the questionnaire (*n* = 94).

The majority of participants began their KD or LC diet at the time of initial diagnosis or during cancer therapy: 55 participants (58.5%) stated that they followed a KD, 20 participants (21.3%) followed an LCHF diet, and 11 participants (11.7%) followed a LOGI diet (Table 2). Twenty eight percent actively restricted their calories in addition to their LC diet during cancer therapy. The remaining eight participants (8.5%) stated that they used some other diet or no specific diet during cancer therapy. We also asked the participants what kind of dietary approach they were following on the day they filled out the questionnaire: 38% were on a KD, 37% an LCHF diet, 19% an LC diet, and 6% followed some other diet (Figure 1). The participants were asked to state for how long they were following one of the LC diets. Sixty-three participants (67%) followed some form of LC diet for more than 1 year, 15 (16.0%) stayed on the diet for 5 – 12 months, 14 stayed on the diet for 1–4 months, and two for <1 month (Table 3).

**Table 2:** Dietary protocols followed by the study participants at the time of cancer diagnosis or therapy.

**Type of diet**	**Number**	**Relative frequency in %**
Ketogenic diet	55	58.5
LCHF (Low Carb High Fat)	20	21.3
Low-Carb (LOGI)	11	11.7
Other	8	8.5
Total	94	100.0

**Table 3:** Adherence to the chosen LC diet among study participants.

**Duration**	**Number of participants**	**Relative frequency**
<1 month	2	2.1%
1–2 months	7	7.4%
2–3 months	5	5.3%
3–4 months	2	2.1%
Longer than 5 months	15	15.9%
Longer than 1 year	63	67%
Total	94	100%

### Individual Perception of Feasibility

An individual's perception of feasibility is an important factor for long-term success. We asked each study participant how easy or difficult it was to implement and adhere to the diet. To get a better understanding of the general cooking skills and to differentiate if and why some may have struggled with the diet, we asked a set of questions regarding cooking and general engagement with the diet.

Seventy-two (77%) participants stated that they enjoy cooking, and 84 (89%) had no difficulties (“easy” or “very easy”) finding appropriate recipes. Participants who stated they did not like to cook tended to rate the diet as being hard to adhere to long term (r_bis_ = −0.201; *p* = 0.053).

**Figure 2:**
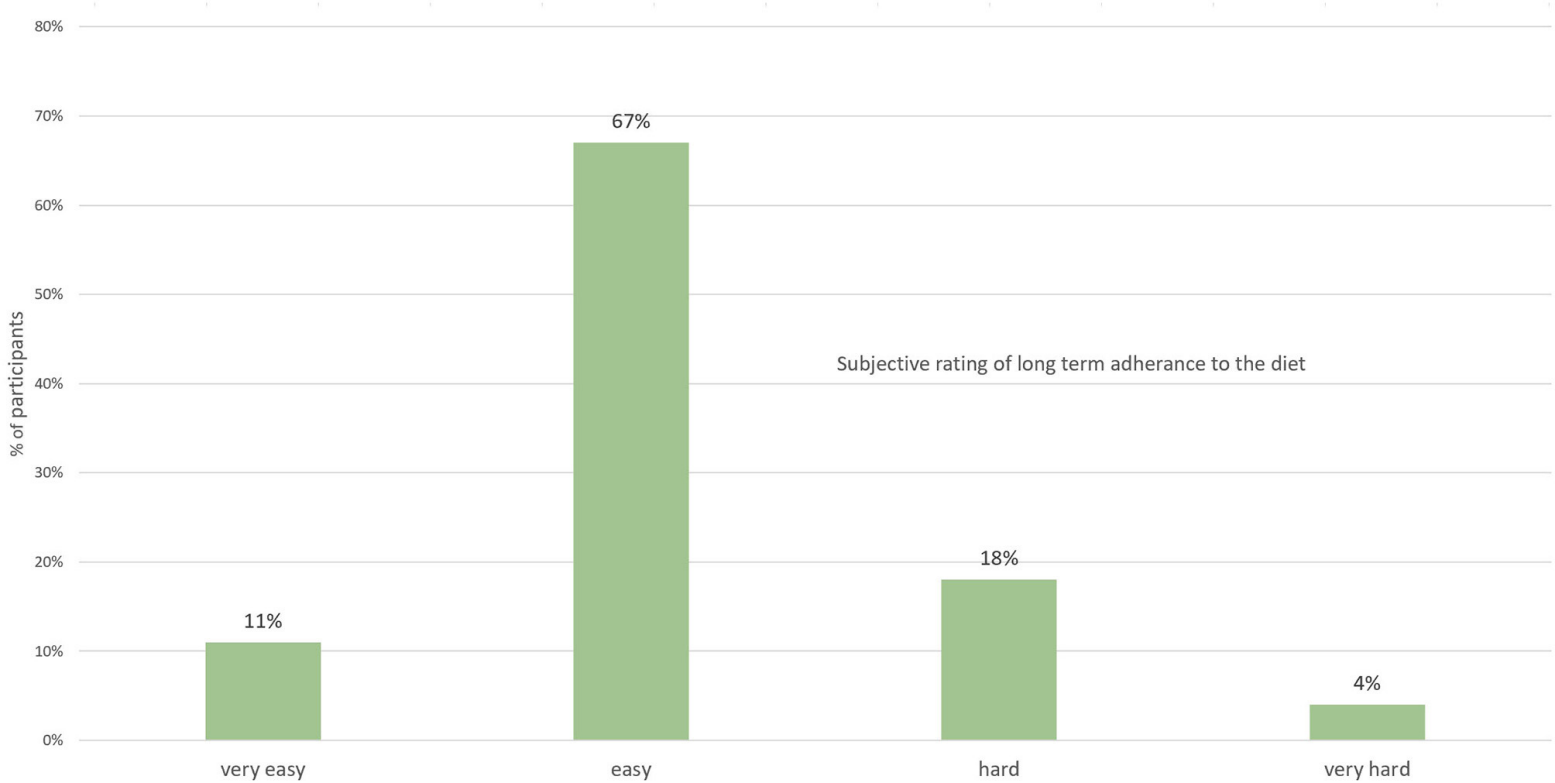
Subjective rating by participants on the difficulty to adhere to the diet long term (*n* = 94).

Overall, 67% found long-term adherence to the diet to be “easy” and 11% described it as “very easy.” On the other hand, 22% described the diet as being “hard”; (18%) or “very hard” (4%) to adhere to long term (Figure 2).

We further analyzed the data using Spearman's rank correlation coefficient to elucidate if there is a correlation between preference for fatty foods and how hard or easy someone found the diet to implement and to adhere to. We found a weak, negative but non-significant correlation (*r* = −0.193; *p* = 0.063). In other words, a person who likes fatty food was more likely to perceive the diet as being easy to adhere to.

### Dietary Advice and Medical and Social Support

Adherence to the diet, professional guidance, as well as social and medical support are important factors that determine the success of any intervention. We asked the study participants if they logged their daily food intake, and 58% responded that they did. Only 18% sought professional support from a dietitian; therefore, 82% implemented the diet on their own, without supervision or counseling. When we asked the participants if they would have liked professional support, 41% responded affirmatively.

**Figure 3:**
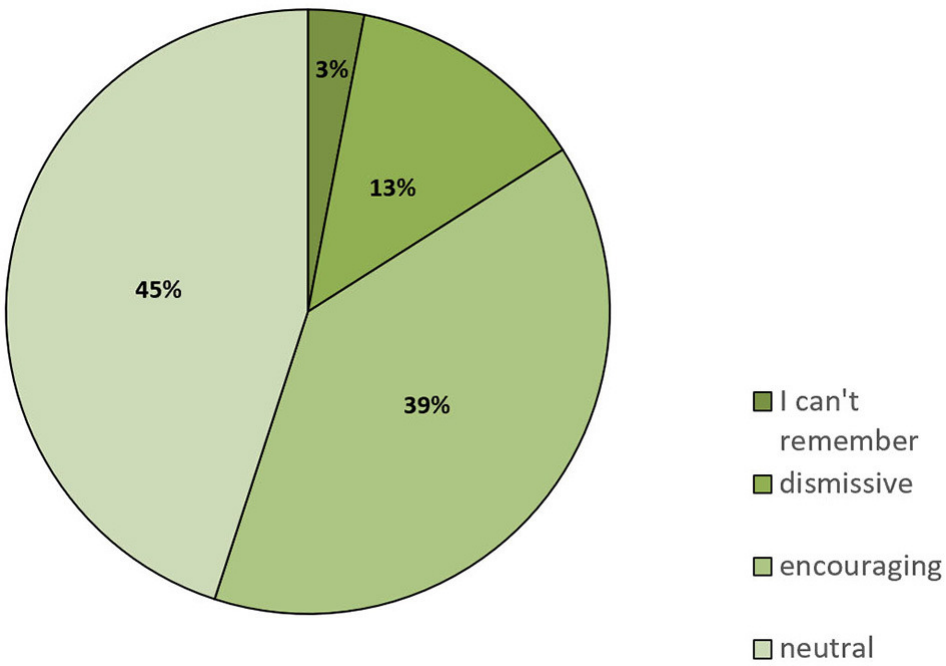
Responses to the question of how family and friends reacted when informed that the patient had switched to a KD or similar LC diet (*n* = 94).

Social support and a close, trusting relationship with their physicians is important for patients. On the one hand, a dietary intervention is much more likely to be continued if it is supported by the patient's family. On the other hand, physicians should be aware of dietary or any other changes made by the patient because this could influence medication or other aspects of therapy. We asked the study participants how their family and friends reacted to their decision to change the diet: 39% stated that family and friends were encouraging, 45% got a neutral reaction, and 13% stated that their family and friends dismissed the idea of a dietary intervention (Figure 3).

**Figure 4:**
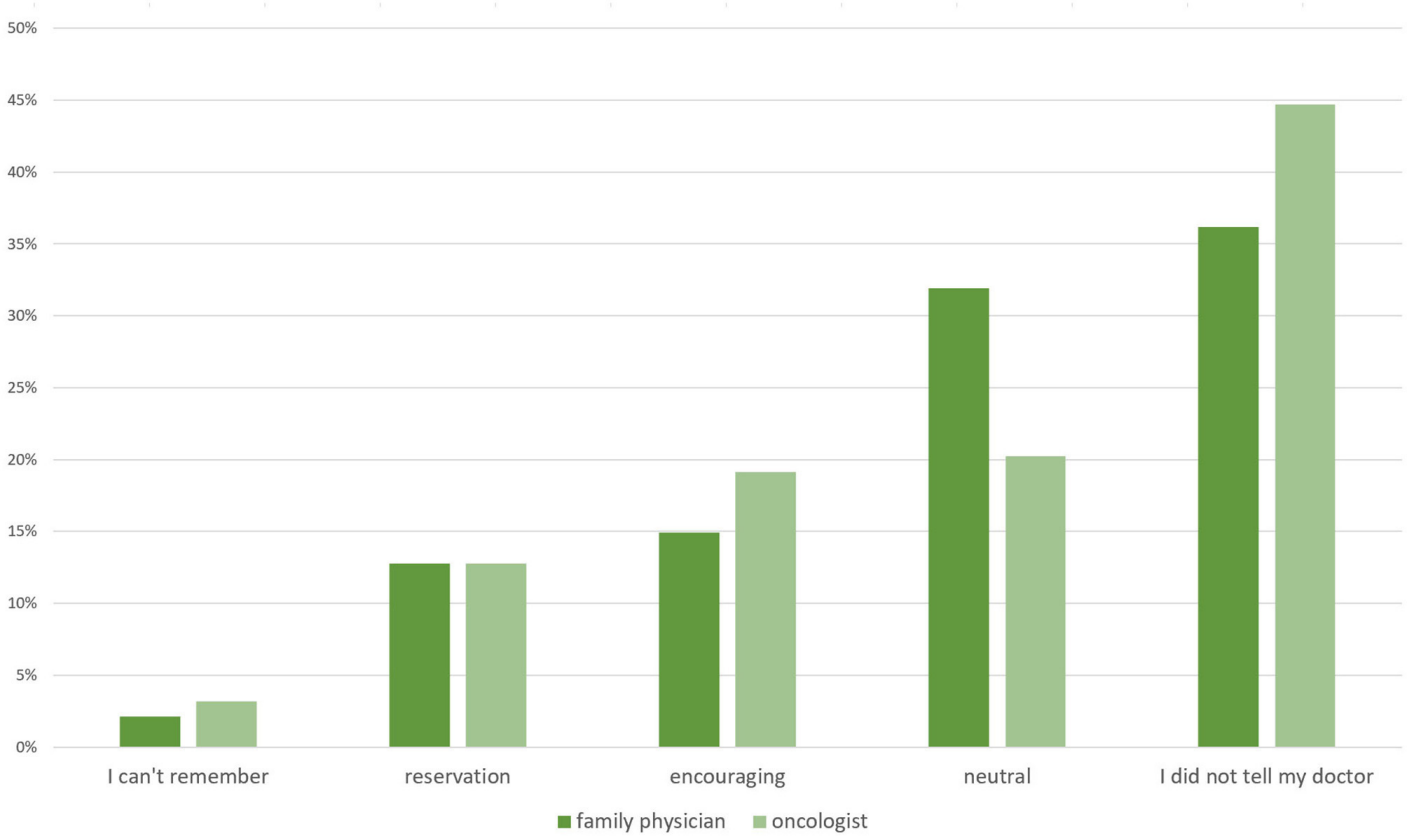
Responses to the question of how their family physicians and oncologists reacted when informed that the patient had switched to a KD or similar LC diet (*n* = 94).

When asked whether they had informed their doctors about their dietary changes, 36% stated they did not tell their family physician and 45% did not tell their oncologist. Approximately 13% responded that their doctors expressed great reservations about the dietary changes. Only 15% of patients stated that their family physician encouraged their decision to change their diet, whereas the percentage of oncologists doing so was slightly higher (19%). Thirty two of family physicians and 20% of oncologists were said to have reacted in a neutral manner (Figure 4).

### Effects of the Diet on Quality of Life and Physical Well-Being

To evaluate effects of the diet on QoL and general physical well-being, we asked the participants a series of questions about the effects of chemotherapy on their ability to perform tasks of everyday life. The study participants were asked if the diet had any influence on side effects of standard cancer therapy, on body weight and if they felt differently because of the dietary changes.

We asked the study participants how they felt chemotherapy affected their ability to cope with tasks of everyday life and if they were limited in their daily activities. Of the 73 participants who answered this question, 47% stated that they felt severely limited or mildly limited, and 16% felt they were not limited at all. Of those who expressed that the diet had an influence on chemotherapy side effects, ~24% reported that side effects from chemotherapy improved because of the diet. Only one individual reported that the diet made them feel weaker.

**Figure 5:**
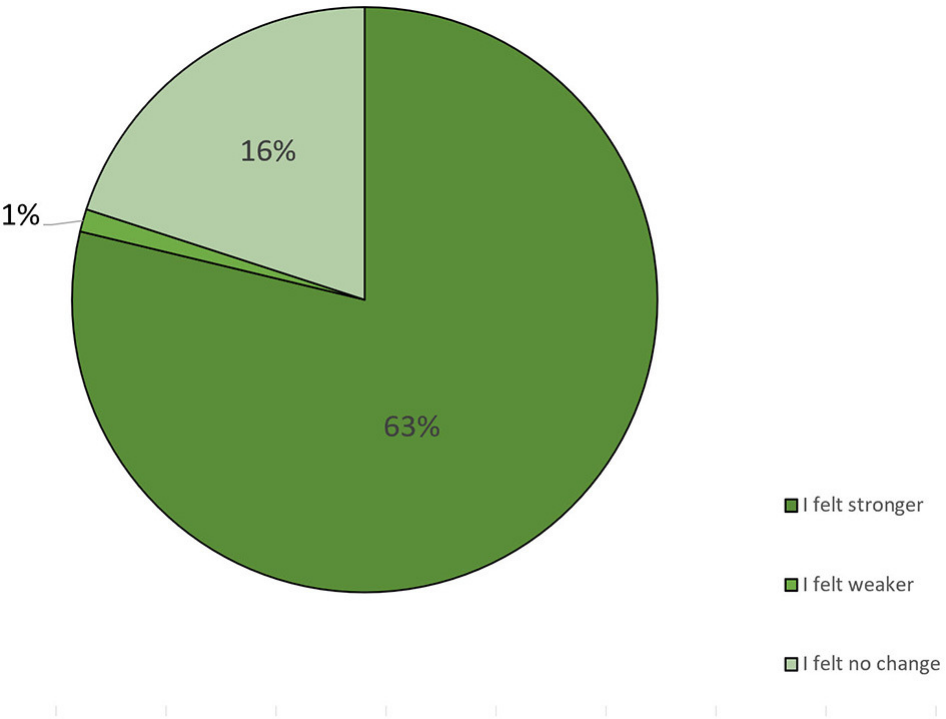
Changes noticed in the quality of life after implementing the diet (*n* = 94).

We asked the participants how they perceived the changes in their well-being after implementing the diet. Of the 94 participants who answered this question, 63% stated that they felt stronger because of the diet, 16% did not feel any change, and 1% stated that they felt weaker because of the diet (Figure 5, Table 4).

**Table 4:** Selected comments from participants indicating which aspects of their QoL improved after they changed their diet.

**Patient characteristics**	**Personal comments**
Cervical-CA; diagnosed 2005	It felt much easier to get moving and to even engage in sport
Thyroid-CA; diagnosed 2011	The extreme tiredness had vanished
Cervical-CA; diagnosed 2012	Working in the garden and social interactions
Breast-CA; diagnosed 2018	Getting up in the morning; less tired; more endurance
Breast-CA; diagnosed 2010	Concentration improved; more energy to meet friends
Breast-CA; diagnosed 2015	General well-being
Breast-CA; diagnosed 2011	Just about everything; not tired anymore
Prostate-CA; diagnosed 2014	I had lost a lot of weight, the KD helped me improve my weight
Sarcoma; diagnosed 2008	Improved energy, mood and motivation
Breast-CA; diagnosed 2013	Side effects disappeared
Leiomyosarcoma; diagnosed 2016	Migraines disappeared, mastering everyday life became easier

### Effects of the Diet on Weight

Cachexia and uncontrolled weight loss are factors that worsen tumor progression and overall prognosis. This is a primary reason why doctors are extremely cautious about dietary changes. On the other hand, overweight and obesity are factors that negatively influence prognosis. Weight loss and weight changes must be evaluated in the context of the starting body weight. At the time of the primary diagnosis, 3.2% of our study participants had been underweight, 52.1% had been of normal weight, 26.6% had been overweight, and 18.1% had been obese. Thirty four percent stated that they had lost some weight during the therapy, 38.3% stated their weight did not change, and ~5% experienced severe weigh loss during cancer therapy. Analyses of the relationship between starting body weight and the extent of weight loss reported by the participants showed a significant correlation between weight loss and severity of overweight (*r* = 0.611; *p* < 0.001). Those who reported the highest weight loss had been overweight or obese before they started the diet.

## Discussion

Changing one's diet in the face of severe illness, often against medical recommendations, is a courageous undertaking. The aim of the present study was to gain insight into the specific characteristics of this group of patients, including their sex, tumor type, food preferences and bodyweight. To our knowledge this is the first study to ask these questions in cancer patients who have been following a KD or LC diet. Largescale clinical studies are extremely expensive and often take years to be published, so an online survey like the one described here can provide valuable insights and help to design further clinical trials.

The preponderance of females (82%) in our study is striking. One possible reason for this could be that women are more active in self-help groups and tend to have more frequent contact with health care professionals. This can be seen in a report compiled by Cancer Research UK, the National Cancer Intelligence Network (NCIN), Leeds Metropolitan University, and the Men's Health Forum (18). This tendency among women to seek help and actively engage in their health was also reflected in our study.

In more than two thirds of the participants in our study, the tumor had not formed metastases at the time of the initial diagnosis. This must be considered in the evaluation of QoL, because the stage of the disease determines how aggressive the standard therapy must be. The most common tumor type reported was breast cancer, followed by colorectal cancer, prostate cancer, melanoma, and lung cancer. The melanoma cases deserve specific attention because, since the work of Xia et al. who reported that ketosis enhances tumor growth of BRAF V600E-mutated melanoma xenografts in nude mice, there has been an intense debate about whether those findings are applicable to humans (19). However, a recent study in immunocompetent mice reported that a KD slowed tumor growth, including melanoma, and also synergized with anti-PD1 therapy (20).

We initially had 5 participants with melanoma. Unfortunately, two participants had to be excluded because instead of providing the date of their initial diagnosis, they provided the date for when they filled out the questionnaire. The remaining three had all been diagnosed with melanoma at least 5 years prior to the study (one in 2012, two in 2013), and all three stated they have been following an LCHF diet for more than 1 year. Two declared that they followed a KD during cancer therapy. These findings in melanoma patients seem promising. This is also in line with a human study, in which the best response to a KD was reported in a patient with BRAF-V600 positive stage IV melanoma (16). In our study, an evaluation of the effect of KDs on tumor progression was not possible, as this would require detailed analysis of clinical data as well as involvement of the physicians and oncologists of the patients, all beyond the scope of the study.

As expected from both the age distribution and the nature of the topic, most participants first read about the KD on the internet and also obtained information on how to implement the diet from there. One reason for this might be that, until recently, there have been very few books written about the KD, especially for non-medical audiences. This publication deficiency has dramatically improved in the last 2 to 3 years, as the quantity of lay literature on the KD has exploded, reflecting the growing interest by the public in the KD. Such massive interest in KDs and LC diets might induce pressure on both health professionals and research institutions to perform more preclinical and clinical studies to provide a scientific basis for dietary counseling of cancer patients (21).

As there is much uncertainty among the lay public about the exact makeup of KDs and the various LC diets, we provided a list of different types of diets to choose from in our questionnaire. Eighty-six participants stated that they followed a KD or some other kind of LC diet. Of those, 55 stated that they followed a KD. However, since only 53% said they measured ketones in some way, it is unclear if the rest of the study participants were in ketosis. On the other hand, it is unclear if and to what extent ketosis is needed to be able to influence QoL and tumor progression. Especially for QoL and mitigation of chemotherapy or radiation therapy side effects, an LCHF diet might be sufficient. Frequently, KDs and LCHF diets are considered to be extremely hard to adhere to, and intolerable as a long-term way of eating. This is in contrast to our data. All but 5 participants stated that they still follow some kind of LC diet. More than half of the participants followed the diet longer than one year, and 15 followed the diet for more than 5 months. A study in epileptic children following the KD for several years reported no adverse side effects and good tolerability (22). Also, recent studies with patients following a KD for up to 2 years reported no severe adverse effects and good tolerability (23, 24).

One of the major criticisms of KDs and LCHF diets in general has been that they are bland, which lowers adherence. However, this complaint might be based on outdated dietary formulations from the early days of the KD, when there were very few ketogenic recipes and cookbooks available. Most of our study participants found long-term adherence to the diet to be “very easy.” One reason some individuals may struggle with a high-fat diet could be an ingrained belief that fatty foods are unhealthy. Results of several meta-analyses do not support the “fat is bad” hypothesis (25–29). A large epidemiological study published in 2017 analyzed the dietary intake of 135,335 individuals across 18 countries. Higher carbohydrate intake was associated with higher risk of total mortality, whereas total fat and individual types of fat were related to lower total mortality, and saturated fat showed an inverse association with stroke. Therefore, the authors concluded that global dietary guidelines should be reconsidered in light of these findings (30). Even though fat's reputation may be about to be rehabilitated, for many the fear of fat is still very real and can be a barrier when trying to implement a KD. We hypothesized that a person who likes fatty foods is more likely to successfully adhere to the diet. Indeed, we found a trend for this in our survey. It also appears to be important that the act of cooking is enjoyed, as nearly three quarters of the study participants stated they like to cook. One limitation of our study is that the participants were not asked about the exact formulation of the diet they followed. We also did not differentiate between different forms of KD in the data analysis. The exact type of diet might influence diet adherence.

Only 18% of our study participants had professional support from a dietitian. Thus, the majority implemented the diet on their own, without dietary supervision or counseling, even though nearly half stated they would have welcomed professional support. It is easier to adhere to a diet plan when there is support, useful information, and practical tips on the diet (31). Currently, this gap in professional information and support is being filled by Facebook groups and self-help forums. Even though those groups can be valuable, the information given there is unfiltered and should not replace professional guidance. There is a need for well-trained personnel capable of educating both patients and medical staff based on clinical evidence, and guiding patients and their families through the implementation of the diet. Indeed, support of peers and strong family backing are critical for success. Almost half of the participants had support from their family and friends.

Interestingly, one third of the study participants did not tell their family physician, and nearly half did not tell their oncologist about their altered diet. The reasoning behind this is the fear of rejection. Twenty nine percent of those who told their physicians about the diet reported experiencing a negative response. Rejection by physicians might be based on a general suspicion regarding the use of alternative adjunct treatments in support of standard therapy. In addition, evidence for the benefit of KDs on tumor progression from clinical studies is unfortunately still missing, and therefore general advice to follow a strict KD in cancer patients cannot yet be provided by physicians and health care providers (8).

Besides the illness itself, the side effects of chemo- or radiation therapy can be extremely debilitating. Common side effects are fatigue, hair loss, infection, nausea and vomiting, nerve and muscle damage, cognitive impairment, and weight changes (32, 33). Some of these side effects severely compromise QoL and can even worsen the prognosis, e.g., excessive weight loss and muscle wasting (34). Therefore, every intervention that eases the side effects of standard therapy must be considered as helpful. Data from small human trials and case reports suggest that KDs attenuate chemotherapy side effects, like nausea and fatigue, and reduce the risk of losing lean muscle mass (35). Also, for those cancer patients whose disease is so far advanced that therapy is no longer an option, improvement of QoL is of utmost importance. Nebeling et al. reported a case of a pediatric patient with advanced stage malignant astrocytoma who exhibited significant clinical improvements in mood and new skill development while on a KD. The patient continued the KD for an additional twelve months, remaining free of disease progression (36). In a study of a KD on QoL in 16 patients with advanced metastatic tumors and without conventional therapeutic options, the patients who finished the 3-month intervention period reported improved emotional functioning and less insomnia, while several other parameters of QoL remained stable. Some worsened, reflecting their very advanced disease (10). In a recent study of 80 patients with locally advanced or metastatic breast cancer and randomly assigned either to a KD or a control group for 12 weeks, the KD group showed higher global QoL and physical activity scores compared to the control group (37). This is in agreement with our study cohort, where the majority of participants stated that they felt stronger through the diet: 28 participants had the impression that the diet had an influence on chemotherapy side effects, and 23 declared that the chemotherapy side effects improved because of the diet. Consistent with this, a recent literature review on KD in cancer patients concluded that KDs lessen the collateral effects of adjuvant chemotherapy (by reducing drug toxicity) and improve QoL compared to patients who follow no diet (21).

Cachexia and uncontrolled weight loss are serious complications which also worsen the prognosis of cancer patients. That is why dietary changes of any kind are often discouraged during cancer therapy. At the other end of the spectrum, overweight and obesity are factors which negatively influence disease outcome (38). An additional aspect of weight and weight loss is body composition and the ratio of fat to fat-free mass. Therefore, weight loss *per se* should not always be avoided, but must be evaluated in the context of each patient's starting body weight. Klement and Sweeney found that a KD during radiotherapy resulted in preservation of lean body mass (17). Body composition was measured with bioimpedance analysis (BIA). BIA is considered an appropriate and reproducible method for assessing fat-free mass and total body water, except in cases of extreme body weight or hydration status (39). We hypothesized that a KD or LCHF diet would be weight-modulating. We found a significant correlation between weight loss and severity of overweight. Those who reported the most weight loss were overweight or obese before they started the diet. In a study of 29 breast cancer patients undergoing curative radiotherapy and who followed a KD based on natural foods, after initial water loss, the KD tended to reduce body weight and fat mass while preserving fat-free and skeletal muscle mass (35).

## Summary and Conclusion

Our online survey identified some key factors that influence the implementation of KDs in cancer patients. The most prominent factors are social support, an affinity to cooking, a proclivity to search for new recipes, and a general preference for fatty foods. Based on our findings, we saw a positive effect on QoL (Table 4) and a modulating effect on weight. We also identified a possible gap between a need for professional counseling and the services currently available. In the future, efforts should be made to invest in nutrition experts who are trained in the KD and other LC diets. To provide patients with the best support possible, it is mandatory to be aware of the factors that influence feasibility and implementation of a KD or LCHF diet in cancer patients and consider all available preclinical and clinical evidence.

## Data Availability Statement

The raw data supporting the conclusions of this article will be made available by the authors, without undue reservation.

## Ethics Statement

The studies involving human participants were reviewed and approved by Donauuniversität Krems ethics committee (EK GZ 26/2015-2018). The patients/participants provided their written informed consent to participate in this study.

## Author Contributions

All authors listed have made a substantial, direct and intellectual contribution to the work, and approved it for publication.

## Conflict of Interest

JT was founder of naehrsinn GmbH (Vienna, Austria) and coach for low-carb and ketogenic diets. The remaining author declares that the research was conducted in the absence of any commercial or financial relationships that could be construed as a potential conflict of interest.

## Publisher's Note

All claims expressed in this article are solely those of the authors and do not necessarily represent those of their affiliated organizations, or those of the publisher, the editors and the reviewers. Any product that may be evaluated in this article, or claim that may be made by its manufacturer, is not guaranteed or endorsed by the publisher.

## Abbreviations

BIA: bioimpedance analysis
KD: ketogenic diet
LCHF: low-carbohydrate high-fat diet
LOGI: low glycemic index diet
QoL: quality of life
PDHD: pyruvate dehydrogenase
BHB: β-hydroxybutyrate.
