# Supplementary material for: Implementation of a Low-Carbohydrate Diet Improves the Quality of Life of Cancer Patients – An Online Survey
Source: Front Nutr. 2021 Aug 11;8:661253. doi: 10.3389/fnut.2021.661253 (PMC8384958; doi:10.3389/fnut.2021.661253)
Supplement: Supplementary file 1 [file Data_Sheet_1.DOCX]

Supplemental Material

## Ketogenic Diet –Questionnaire English

Thank you very much for your support!

Thank you for taking the time. This study would not be possible without your support. You are making an important contribution to science. If you wish, you can leave us a personal message in the following text box.

Year of birth

Gender

- - male
  - female
  - other

## Data on your disease

- Date of the initial diagnosis

Format: MM/DD/YYYY

- Tumor type

- Tumor stage (at the time of diagnosis)

- In which organ or in which body part was/is the tumor located at the initial diagnosis?

- Had the tumor already formed metastases at the initial diagnosis?
  - Yes
  - No
- Is/was the tumor PET positive?

Was the tumor visible on a PET Scan?

- - Yes
  - No
  - I did not have a PET Scan
- In which stage of therapy are you currently in (chemo, radiation, etc.)?
  - I have just started the therapy.
  - I am in the middle of the first treatment series / I have just finished it.
  - I have already completed more than one treatment cycle.
  - I finished the therapy more than half a year ago.
  - I finished the therapy more than 1 year ago.
- Which therapies were/are used?

(chemotherapy, radiotherapy, biological, etc.)

- Which chemotherapeutics do/did you get?

- How many treatment cycles were conducted?

- Which other medicines do/did you take during the cancer treatment?

- Was there a surgical intervention in the course of the disease?
  - Yes
  - No
- Do/did you take dietary supplements?
  - Yes
  - No
- Do you have one or more chronic or long-lasting diseases?

Chronic diseases are long-lasting diseases that require constant treatment and control, such as diabetes, autoimmune diseases or cardiovascular diseases.

- - Yes
  - No
  - I don't know
- How did you find out that a dietary change might be helpful for you?
  - Newspaper article
  - TV report
  - Internet / blog article
  - Friends/ family members/ acquaintances called it to my attention
  - Book
  - I can't remember
  - Other
- Which was the diet of your choice during the cancer treatment?
  - Ketogenic diet
  - Paleo diet
  - Atkins diet
  - LCHF (Low Carb High Fat)
  - Low-Carb (LOGI)
- How did you find out about this diet?

Newspaper article, TV report, Internet, friends or acquaintances, books, etc.

- Did you purposely reduce calories during the cancer treatment or do you currently restrict calories purposely?
  - Yes
  - No
- Which diet do you follow now?

Even if you changed between the different diets listed here, please choose the one which you mainly follow.

- - Ketogenic diet
  - Paleo diet
  - Atkins diet
  - LCHF (Low Carb High Fat)
  - Low-Carb (LOGI)
- When did you start the diet change?
  - Immediately after the diagnosis
  - In the first week of the therapy
  - A few months after therapy start
  - After completing the therapy
  - I can't remember
- How long did you follow the diet?
  - Less than 1 month
  - 1 - 2 months
  - 2 - 3 months
  - 3 - 4 months
  - More than 5 months
  - More than 1 year
- Did you test for ketones?
  - Yes
  - No
- Why did you decide not to follow the diet anymore?

Fill out only if this applies to you.

- How hard was it/is it for you to implement the diet you opted for during the cancer therapy?

- How hard is it in your opinion to implement the diet in the long term?

- Do you like to cook?
  - Yes
  - No
- How hard was it/is it to find appropriate recipes?
  - very easy
  - fairly easy
  - hard
  - very hard
  - I can't say
- How hard was it/is it to find appropriate ingredients/ food?
  - very easy
  - fairly easy
  - hard
  - very hard
  - I can't remember
- Do you wish there were more ready-made products to make it easier to follow the diet?
  - Yes
  - No
  - No opinion
- Did/do you keep a food diary?
  - Yes
  - No

## Questions on supervision

- Did you have the support of a dietician in terms of diet change?
  - Yes
  - No
  - I can't remember
- Where and how did you find out about the diet?
  - Books
  - Facebook groups
  - Internet forums
  - Blogs/ Websites
  - Lectures/ seminars
  - Other
- Can you remember which source of information was especially helpful?

Please list books, websites, authors or bloggers who helped you learn more about the diet.

- How did your family/ social environment react to your change of diet?
  - neutrally
  - negatively
  - encouragingly
  - I can't remember
- How did your family doctor react to your change of diet?
  - neutrally
  - negatively
  - encouragingly
  - I haven't told my family doctor about my diet change
  - I can't remember
- How did your treating oncologist react?
  - neutrally
  - negatively
  - encouragingly
  - I haven't told the oncologist that I changed my diet
  - I can't remember
- What is the current status of your cancerous disease?

## Quality of life

- To what extent are you/were you limited by the chemotherapy in your daily activities?
  - Substantially limited
  - Limited, but not substantially
  - Not limited
  - I can't remember
- Did the side effects change due to the diet?
  - Yes
  - No
  - I can't remember
- Due to the diet change, I felt ...
  - stronger
  - weaker
  - unchanged
- Which activities did you find easier after you started the diet?

- Which activities did you find harder after you started the diet?

- How was your weight at the beginning of the disease?
  - underweight
  - normal weight
  - slight overweight
  - severe obesity (adiposity)
- How did your weight change during the therapy?
  - My weight did not change
  - I lost hardly any weight
  - I lost some weight
  - I lost a lot of weight
- Did your weight change due to the diet change?
  - My weight did not change
  - I lost hardly any weight
  - I lost some weight
  - I lost a lot of weight
- My doctor told me that as a treatment-related side effect, my weight would ...

Certain medicines and chemotherapeutics can affect weight.

- - not change.
  - probably increase.
  - probably decrease.
  - I can't remember.

## Personal details

- Which is your highest qualification attained?

(e.g. high school, college, university, professional training, etc)

- Are you currently employed?
  - Full-time employed
  - Part-time employed
  - Marginally employed
  - Temporarily exempted
  - Not employed
- How many hours do you work a week?

- How many people live in your household?
  - I live alone
  - 2
  - 3
  - 4
  - more than 4 people
- What is your marital status?
  - Married, cohabiting with partner
  - Married, separated from partner
  - Single
  - Divorced
  - Widowed

## How often do you eat the following meals?

- Breakfast
  - Daily
  - Often, 4-6 times a week
  - Occasionally, 2-3 times a week
  - Rarely, 1-4 times per month
  - Never
- Snack
  - Daily
  - Often, 4-6 times a week
  - Occasionally, 2-3 times a week
  - Rarely, 1-4 times per month
  - Never
- Lunch
  - Daily
  - Often, 4-6 times a week
  - Occasionally, 2-3 times a week
  - Rarely, 1-4 times per month
  - Never
- Coffee and cake
  - Daily
  - Often, 4-6 times a week
  - Occasionally, 2-3 times a week
  - Rarely, 1-4 times per month
  - Never
- Dinner
  - Daily
  - Often, 4-6 times a week
  - Occasionally, 2-3 times a week
  - Rarely, 1-4 times per month
  - Never

## How often do you drink the following beverages?

- Coffee
  - Daily
  - Often, 4-6 times a week
  - Occasionally, 2-3 times a week
  - Rarely, 1-4 times per month
  - Never
- Tea
  - Daily
  - Often, 4-6 times a week
  - Occasionally, 2-3 times a week
  - Rarely, 1-4 times per month
  - Never
- Fruit juices
  - Daily
  - Often, 4-6 times a week
  - Occasionally, 2-3 times a week
  - Rarely, 1-4 times per month
  - Never
- Soft drinks (Coca Cola, Fanta, Ice tea, lemonades etc.)
  - Daily
  - Often, 4-6 times a week
  - Occasionally, 2-3 times a week
  - Rarely, 1-4 times per month
  - Never
- Wine
  - Daily
  - Often, 4-6 times a week
  - Occasionally, 2-3 times a week
  - Rarely, 1-4 times per month
  - Never
- Beer
  - Daily
  - Often, 4-6 times a week
  - Occasionally, 2-3 times a week
  - Rarely, 1-4 times per month
  - Never
- Mineral water
  - Daily
  - Often, 4-6 times a week
  - Occasionally, 2-3 times a week
  - Rarely, 1-4 times per month
  - Never
- Tap water
  - Daily
  - Often, 4-6 times a week
  - Occasionally, 2-3 times a week
  - Rarely, 1-4 times per month
  - Never

## How often do you eat the following foods?

The questions on bread, pastry and sweets refer to conventional products, NOT to low-carb foods.

- Salad
  - Daily
  - Often, 4-6 times a week
  - Occasionally, 2-3 times a week
  - Rarely, 1-4 times per month
  - Never
- Vegetables
  - Daily
  - Often, 4-6 times a week
  - Occasionally, 2-3 times a week
  - Rarely, 1-4 times per month
  - Never
- Fruits
  - Daily
  - Often, 4-6 times a week
  - Occasionally, 2-3 times a week
  - Rarely, 1-4 times per month
  - Never
- Eggs
  - Daily
  - Often, 4-6 times a week
  - Occasionally, 2-3 times a week
  - Rarely, 1-4 times per month
  - Never
- Bread
  - Daily
  - Often, 4-6 times a week
  - Occasionally, 2-3 times a week
  - Rarely, 1-4 times per month
  - Never
- Meat
  - Daily
  - Often, 4-6 times a week
  - Occasionally, 2-3 times a week
  - Rarely, 1-4 times per month
  - Never
- Fish
  - Daily
  - Often, 4-6 times a week
  - Occasionally, 2-3 times a week
  - Rarely, 1-4 times per month
  - Never
- Pasta, rice, dumplings or potatoes
  - Daily
  - Often, 4-6 times a week
  - Occasionally, 2-3 times a week
  - Rarely, 1-4 times per month
  - Never
- Sausage, ham or bacon
  - Daily
  - Often, 4-6 times a week
  - Occasionally, 2-3 times a week
  - Rarely, 1-4 times per month
  - Never
- Dairy products (milk, yoghurt, cheese, cream)
  - Daily
  - Often, 4-6 times a week
  - Occasionally, 2-3 times a week
  - Rarely, 1-4 times per month
  - Never
- Sweets (cakes, biscuits, chocolate, gummy bears etc.)
  - Daily
  - Often, 4-6 times a week
  - Occasionally, 2-3 times a week
  - Rarely, 1-4 times per month
  - Never
- Nuts
  - Daily
  - Often, 4-6 times a week
  - Occasionally, 2-3 times a week
  - Rarely, 1-4 times per month
  - Never
- Ready meals
  - Daily
  - Often, 4-6 times a week
  - Occasionally, 2-3 times a week
  - Rarely, 1-4 times per month
  - Never
- Do you use sweeteners?
  - Yes, daily
  - Yes, sometimes
  - Rarely
  - Never
- Which sweeteners do you use?

Please list all sweeteners and sugar substitutes that you use, even if you use them only rarely. You can also write the brand name of the product you use.

## How much did you enjoy the following foods before you started the diet?

- Fatty dishes

(roast pork, bacon, sausage products, fried food, lard, etc.)

- - A lot
  - Gladly
  - Somewhat
  - Not at all
- Fatty sauces

(mayonnaise, Hollandaise sauce, creme fraiche, cream sauce, etc.)

- - A lot
  - Gladly
  - Somewhat
  - Not at all
- Dairy products and dishes with cream

(cheese, butter, cream sauce, cream, Mascarpone, etc.)

- - A lot
  - Gladly
  - Somewhat
  - Not at all
- Cakes, desserts, chocolate and other sweets
  - A lot
  - Gladly
  - Somewhat
  - Not at all
- Bread, rolls, pastry
  - A lot
  - Gladly
  - Somewhat
  - Not at all
- Meat and fish
  - A lot
  - Gladly
  - Somewhat
  - Not at all

## Closing questions

- May we contact you?

Would you agree to be contacted by us for a follow-up study?

- - Yes
- Did we forget any question?
